# Reading comprehension and strategy use: Comparing bilingual children to their monolingual peers and to bilingual adults

**DOI:** 10.3389/fpsyg.2022.986937

**Published:** 2022-11-24

**Authors:** Deanna C. Friesen, Katherine Schmidt, Taninder Atwal, Angela Celebre

**Affiliations:** Faculty of Education, University of Western Ontario, London, ON, Canada

**Keywords:** bilingualism, reading comprehension, reading strategies, children, English language learner

## Abstract

The current study investigated the predictive ability of language knowledge and reported strategy use on reading comprehension performance in English-speaking monolingual and bilingual students. One hundred fifty-five children in grade 4 through 6 (93 bilinguals and 62 monolinguals) were assessed on receptive vocabulary, word reading fluency, reading comprehension, and reading strategy use in English. An additional 38 adult bilinguals (i.e., English Language Learners) were assessed on the same measures. Although, the bilingual adult group and bilingual children had significantly lower English vocabulary knowledge relative to the monolingual children, the bilingual adults exhibited reading comprehension performance that was on par with the monolingual children; both groups outperformed the bilingual children. This discrepancy was accounted for by reported strategy use, wherein bilingual adults reported more inferencing, more connecting between sections of text and more reference to the text structure than the children. Reported strategy use also accounted for unique variance in reading comprehension performance above and beyond the contributions of English vocabulary knowledge and word reading fluency. Findings highlight the strategies that successful readers report and emphasize the value of promoting effective strategy selection in addition to language instruction in the development of reading comprehension skill.

## Introduction

By definition, bilinguals divide their language exposure between two languages. Consequently, they have fewer opportunities to develop proficiencies in each language, and often exhibit weaker second language reading comprehension performance than native speakers of that language (e.g., Aarts and Verhoeven, 1999; Geva and Farnia, 2012; Raudszus et al., 2021). However, reading comprehension success also depends on deploying strategies to extract meaning from print (McNamara, 2012). For bilinguals, it may be especially important to use strategies to offset weaker second language (L2) knowledge (Kolić-Vehovec and Bajśanski, 2007). In the current study, we investigated how three groups of readers reported their use of reading comprehension strategies in English and whether this reported strategy use predicted reading comprehension success.

Unfortunately, the reading comprehension achievement gap between monolingual readers and L2 readers can widen throughout elementary school (e.g., Droop and Verhoeven, 2003; Farnia and Geva, 2013; Raudszus et al., 2021). In Canada, Farnia and Geva (2013) reported that unlike their monolingual peers, the growth trajectory for L2 learners' reading comprehension performance leveled off from Grade 4 to 6. Droop and Verhoeven (2003) also reported stronger reading comprehension in monolinguals relative to their bilingual peers from grade 3 to 4 in the Netherlands. These differences may be problematic given the importance of reading comprehension for both school and career success (August and Shanahan, 2006). Key then, is to understand the locus of these reading comprehension differences and provide instruction to address students' literacy needs.

The predominant approach to understanding reading comprehension is to examine the relative contributions of component skills. Arguably, the Simple View of Reading model (SVR; Hoover and Gough, 1990) is the most widely cited framework of reading comprehension development. In the SVR model, reading comprehension is the product of decoding ability and linguistic comprehension (D x LC = RC). Decoding refers to word recognition processes (e.g., using grapheme-phoneme correspondences), whereas linguistic comprehension refers to the skills necessary to understand language (e.g., vocabulary, syntax, grammar, discourse processes). Several studies have confirmed the importance of both language knowledge and decoding ability for successful reading comprehension (See Castles et al., 2018 for a review). Indeed, reading comprehension success is unlikely if one of these components is missing or weak (Joshi and Aaron, 2000).

Differences in linguistic comprehension have been isolated as the main source of reading comprehension language group differences. In Droop and Verhoeven (2003), bilinguals exhibited faster word decoding than their monolingual peers but poorer language proficiency and reading comprehension performance. Likewise, Geva and Farnia (2012) reported no word reading differences between groups despite weaker reading comprehension performance and syntax knowledge in English second language learners. Raudszus et al. (2021) found that L2 readers with high vocabulary knowledge showed comparable reading comprehension growth relative to high vocabulary L1 readers. In contrast, L2 readers with low vocabulary exhibited less reading comprehension growth than monolingual readers who also had weak vocabulary knowledge. Importantly, Bialystok et al. (2010) have reported a consistent 9-point difference on a standardized English vocabulary measure between monolinguals (*N* = 966) and bilinguals (*N* = 772) who were between the ages of 3 and 10 years old in Canada. Such findings support Droop and Verhoeven's proposal that group differences in reading comprehension are affected by language knowledge in a top-down fashion, as opposed to bottom-up word decoding.

Importantly, as age increases, language knowledge becomes a better predictor of reading comprehension success than decoding ability (Gough et al., 1996; Storch and Whitechurst, 2002; Proctor et al., 2006; Gunnerud et al., 2022). Proctor et al. (2005) found that with sufficient L2 decoding ability, L2 vocabulary is the critical variable for L2 reading comprehension outcomes. For adults, decoding ability is often not a predictor of reading comprehension performance (e.g., Landi, 2010; Friesen and Frid, 2021). Babayigit and Shapiro (2020) reported that both English vocabulary and grammar knowledge are strong predictors of English reading comprehension performance and should be strongly targeted for L2 instruction. A meta-analysis demonstrated that several studies report that language comprehension skills are stronger predictors of reading comprehension in the L2 than in the L1 (Melby-Lervåg and Lervåg, 2014). Taken together, these findings indicate that these language skills need to be supported and/or offset in bilingual students' education.

Although language variables have received the most attention, more recent work has expanded the scope of reading comprehension predictors to include cognitive (e.g., working memory; Farnia and Geva, 2013), affective (e.g., motivation, e.g., Cho et al., 2019) and meta-cognitive measures (e.g., van Steensel et al., 2016). In their Reading Systems Framework, Perfetti and Stafura (2014) identify knowledge (e.g., vocabulary), processes (e.g., decoding) and cognitive abilities (e.g., executive functions) as reader variables that underlie reading comprehension success. However, reading is dynamic, and individuals modify their reading behaviors as a function of the nature of the text and their reading goals (Rand Reading Study Group, 2002). Presumably then, effective strategy selection in response to the reading demands is also a critical ability for consistent reading comprehension success.

The current study examined whether the type of strategies used by L2 readers and monolinguals predict reading comprehension success beyond what is accounted for by traditional language measures. We focused on strategy use for several reasons. First, as noted by Afflerbach et al. (2008), reading strategies are “deliberate, goal-directed attempts to control and modify the reader's efforts to decode text, understand words and construct meanings of texts” (p. 368). Thus, they are subject to explicit instruction from teachers and strategies can be targeted for improvement. Second, reading comprehension strategy instruction is integral to most language arts curriculums. However, strategies are often listed without identifying which strategies may work together or be most effective to retain content (e.g., Ontario Ministry of Education, 2006). Finally, a meta-analysis found that L2 strategy instruction produces small to moderate effect sizes and that several variables impact the strength of the outcomes, including strategy selection itself (Plonsky, 2011). Here, our focus is on identifying reading comprehension strategies that are correlated with reading comprehension success to help inform effective strategy selection.

Reading strategies have been categorized in several ways (e.g., Mokhtari and Reichard, 2004; Plonsky, 2011). Here we focus on strategies that can be used during reading. For example, Block (1986) divided strategies into general and local strategies. General strategies included prediction, identifying text structure, questioning, and using background knowledge. Local strategies pinpoint particular parts of texts and include paraphrasing, rereading, questioning the meaning of a clause or a sentence, and questioning the meaning of vocabulary. Janzen and Stoller (1998) also identified a set of strategies which included predicting, asking questions, checking predictions, or looking for answers to questions, connecting the text to the prior knowledge, summarizing, connecting within the text, and recognizing text structure. The current study adopted the approach of looking at several of these individual strategies (see Supplementary material) because these strategies are often emphasized in both curriculum documents (Ontario Ministry of Education, 2006) and consequently, in language classrooms.

In the monolingual literature, higher-order processes have been found to predict reading comprehension success. For example, Oakhill et al. (2003) followed monolingual children from ages 7–10. Inferential ability, comprehension monitoring ability and knowledge of text structure at age 7 and 8 predicted reading comprehension success at age 10. Good comprehenders also analyze arguments found in text, utilize background knowledge (Saricoban, 2002), and pose questions (Yopp, 1988). Engaging in visualization has also been shown to result in reading comprehension gains (Pressley, 2000; Erfani et al., 2011). Importantly, poor language proficiency may be offset by engaging in some of this effective strategy use (Carrell, 1989; Padrón, 1992; Kolić-Vehovec and Bajśanski, 2007; Friesen and Haigh, 2018).

Effective reading strategy use in L2 has been studied using both questionnaires (e.g., Mokhtari and Reichard, 2004; Afsharrad and Benis, 2017; see Friesen and Frid, 2021 for a brief review) and think-aloud protocols (e.g., Jiménez et al., 1996; Chamot and El-Dinary, 1999; Park and Kim, 2015). Work with adults has typically favored using questionnaires. Although questionnaire data is easier to collect, data is based on respondents' reflections of their strategy use and may not be accurate (Brown, 2017). These retrospective meta-cognitive processes may also be beyond the capabilities of young readers to evaluate. We favor the think-aloud procedure where readers report their thought processes during reading because rich descriptive data is captured online (Chamot and El-Dinary, 1999). Although there are concerns that readers are only reporting a subset of their strategy use and that comprehension may be altered, think-alouds do enable insight into the types of strategies a reader is able to access during online processing.

In a literature review, Brantmeier (2002) summarized that successful L2 adult readers prefer top-down strategies such as integrating distinct parts of text, referring to text structure, and making links to background knowledge, whereas less successful readers used more bottom-up strategies such as rereading and identifying lexical problems. In a think-aloud study, Lin and Yu (2015) found that more proficient L2 adults engaged in more effective and varied strategies that were aimed toward comprehension in their L2, whereas less proficient L2 users were focused on language-oriented strategies. More proficient L2 readers asked more questions, paraphrased more, translated more, and used more contextual cues than the less proficient bilingual readers.

A few think-aloud studies have been conducted with children as L2 readers. One main concern with this work is that sample sizes are often small. For example, Park and Kim (2015) examined strategy use with four L2 learners of English in Grade 4 or 5. Students used a dialogic approach where students spoke aloud to others to engage in meaning-making. They posed questions, made inferences, relied on previous knowledge, and drew conclusions. In another example, Jiménez et al. (1996) compared eight good L2 readers with three poor L2 readers on their think-aloud strategies. Good L2 readers translated text, resolved unknown vocabulary, monitored comprehension, connected text to previous knowledge and made inferences/conclusions. Poor L2 readers identified unknown words but did not attempt to determine their meaning. In García and Godina's (2017) work, Grade 5 bilinguals with more L2 proficiency used more varied strategies, generated more plausible inferences, referred to background knowledge, paraphrased, and monitored comprehension more than the students with less L2 proficiency. Although these studies are informative, it is not clear whether the strategies explained unique variance in reading comprehension performance in bilingual readers that was not accounted for by their language abilities.

Work by Frid and Friesen (2020) examined which reading strategies were related to reading comprehension success in French Immersion students in Grades 4 and 5. These students present a unique population since English is L1, but L1 reading instruction begins in Grade 4. Participants predicted and generated inferences more in L1. They summarized and referred to unknown vocabulary more in L2 than L1. Additionally, reliance on inferencing behaviors and text analysis accounted for unique variance in reading comprehension performance in each language. A similar emphasis on these strategies was reported by Friesen and Frid (2021) in a study that addressed the same questions in English-French bilingual adults.

### The current study

The current study asked (1) whether strategy use differed as a function of language experience by comparing performance across three groups of English speakers and (2) whether strategy use accounted for unique variance in the reading comprehension performance beyond language knowledge (i.e., receptive vocabulary and word reading fluency). To our knowledge, this is the first study to compare L2 children with both age-matched monolingual children and language-matched L2 adults. The monolingual children and bilingual children differed in their English vocabulary knowledge, enabling us to draw conclusions about the role of language in reading comprehension performance. In contrast, the bilingual adults had the same degree of English vocabulary proficiency as the bilingual children but differed in their reading experience from both groups of children, enabling us to examine the role of greater literacy expertise in reading comprehension performance. By making these comparisons, we can gain insight into the importance of both language proficiency and reading experience on reading comprehension performance and strategy use.

Both language measures and strategy use were assessed and used as predictors of reading comprehension performance. Think-aloud reponses were coded for ten strategies (i.e., reference to vocabulary, reference to text structure, reference to background knowledge, connecting to texts or previous think-alouds, summarizing, necessary inferences, elaborative inferences, questioning, predicting, and visualizing). Necessary inferences were drawn conclusions required to maintain text cohesion. Elaborative inferences were reasonable conclusions based on the text but unnecessary for understanding (see Supplementary material for full descriptions and examples). We asked whether monolingual English readers and English second language readers reported similar reading strategies to construct meaning from text. We also asked whether individual reading strategies and language abilities uniquely account for reading comprehension success in all three groups. Ideally, knowledge about how second language learners process text will serve to support reading comprehension development in struggling readers.

## Method

### Participants

Participants were 155 students in Grades 4 to 6 from a large school board in Ontario, Canada. Of these, 93 students were bilingual with English as one of their languages (*M*_age_ = 10.6, *SD* = 1.0; 52 females) and 62 students were English monolingual speakers (*M*_age_ = 10.7, *SD* = 1.0; 37 females). To be classified as bilingual, students had to speak a language other than English in the home; English was learned either both in the home and school, or just in school. On average, bilingual children had 4.5 years (*SD* = 1.9) of English schooling compared to 5.7 years (*SD* = 1.0) for monolingual children. Home languages of the bilinguals were Albanian (2), Amharic (1), Arabic (42), Bengali (1), Bosnian (2), Chinese (12), Dari (1), Hindi (1), Khmer (1), Korean (7), Kurdish (4), Pashto (2), Portuguese (1), Punjabi (2), Russian (1), Sindhi (1), Tagalog (1), Tamil (1), Turkish (1), and Urdu (5). Parents reported that bilingual children spent an average of 5.0 (*SD* = 7.2) hours reading in English outside of school per week, whereas monolingual children spent an average of 7.2 (*SD* = 13.8) hours per week. Bilingual children read for an average of 2.1 h (*SD* = 4.2) in their other language. Parents reported that their child preferred to read in English [bilinguals: 4.4 (*SD* = 0.98), monolinguals: 4.5 (*SD* = 1.4)] on a five-point scale where 5 was strongly agree (see Table 1 for additional demographic information).

**Table 1:** Means and standard deviations (in parentheses) for background and language measures for each group.

**Measures**	**Monolingual children**	**Bilingual children**	**Bilingual adults**
**Questionnaire measures**
English AoA (in years)	–	3.5 (3.0)^a^	8.8 (2.7)
Other language proficiency rating^b^	–	7.6 (2.6)	8.9 (2.6)
Language use (speaking)^c^	1.0 (0.1)	4.2 (2.0)	3.5 (1.5)
Language use (reading)^c^	1.0 (0.1)	2.0 (1.3)	1.8 (1.2)
**English language measures**
Receptive vocabulary (max. 204)	141.0 (22.3)^d^	114.6 (37.5)	119.1 (21.0)
Word reading fluency (max. 167)	103.0 (25.4)	96.8 (30.8)	103.3 (20.2)
Reading comprehension (max. 24)	12.3 (4.4)	9.5 (5.6)	12.1 (2.7)

a Participants tested in the first cohort are missing this data (~ a third of participants).

b Rating scale from 1 to 10 (where 1 is poor & 10 is native-like).

c Rating scale from 1 to 7 (where 1 was all English and 7 was all other language).

d As a point of comparison, 141 is equivalent to a standard score of 106 based on monolingual norms.

An additional 38 sequential bilingual adults also participated (*M*_age_ = 25.2, *SD* = 3.7; 36 females). Adult participants were all born outside of Canada and had been in Canada for an average of 9.7 months (*SD* = 3.7). First languages included Chinese (35), Farsi (1), Malay (1), and Persian (1). Participants were completing a graduate degree (35 were enrolled in a Teaching English to Speakers of Other Languages program). Participants reported reading in English an average of 18.4 h per week (*SD* = 12.5) and in their first language an average of 14.6 h (*SD* = 10.5) per week (see Table 1 for additional demographic information).

### Measures

#### Language experience questionnaires (parent version & adult version)

The parental questionnaire included rating scales for their child's understanding and speaking ability in their non-English language, questions about language use in the home, and about the child's reading preferences. The adult questionnaire asked participants about language dominance, language proficiency and language use. Both questionnaires also asked about the Age of Acquisition for English (AoA) defined as when the participant started learning the language. Note the scales for proficiency and language use were different in each questionnaire and have been transformed to be on the same scale for ease of interpretation in Table 1.

#### Receptive English vocabulary

Receptive vocabulary was assessed using the Peabody Picture Vocabulary Test (PPVT-III: Form A; Dunn and Dunn, 1997). The test is designed for ages 2.5–90+ years old. In each trial, four images were presented, and an auditory word was heard. Participants selected the picture that matched the word. Items are ordered by word difficulty and standard administration follows basal and ceiling rules. Within the sample, the Spearman-Brown Split-test coefficient was 0.99 for the children and 0.96 for the adults. Raw scores were used rather than standard scores, since absolute vocabulary knowledge is appropriate to make group comparisons and for regression analyses.

#### Word reading fluency

English word reading fluency was assessed using the Test of Word Reading Efficiency (TOWRE; Torgesen et al., 1999). The TOWRE includes sight word reading efficiency (104 words) and phonemic decoding efficiency (63 pseudo-words). Participants read as many items as possible in 45 s for each subtest. Inter-rater reliability was calculated on a subset of participants (25% of child data and 38% of adult data). For the children, agreement was 0.98 on the words and 0.92 on the non-words. For the adults, agreement was 0.98 and 0.91, respectively. The raw total for both measures were added together and was used in the analyses as a measure of word reading fluency.

#### Reading comprehension and strategy use task

Reading comprehension and strategy use were assessed using four texts taken from the Gray Oral Reading Test (GORT−4th Edition, Form B, Wiederholt and Bryant, 2001). Standard administration of the GORT-4 involves the individual reading texts aloud and responding to multiple-choice questions. Entry points are determined based on an individual's age. Basal and ceiling rules are applied for both fluency and comprehension. Since our focus was not on reading fluency but strategy selection, standard administration was not employed; participants read texts silently. The reason GORT-4 texts were selected is because they provided 14 developmentally sequenced texts for ages 6–18 years. Texts 6, 7, 8, 9, were selected as being age-appropriate for Grades 4 through 8. Text 5 (~ grade 3 level) was used as an exemplar. The texts averaged 119.8 words (*SD* = 25.4) and 8.25 sentences (*SD* = 1.3) each. Two texts were narrative (i.e., one about a turtle on an adventure & one about shipwrecked siblings) and two were expository (i.e., one about problems faced by farmers & one about Harriet Tubman). The range of texts were selected to address concerns about floor/ceiling effects within any one group. Importantly, to compare strategy use and reading comprehension performance directly among groups and avoid confounds based on the materials, the same texts were employed for all participants. Given this decision, a valid concern is how students' language proficiency impacts their performance. This concern was addressed by measuring word reading fluency and receptive vocabulary knowledge, and then accounting for them in the analyses of reading comprehension performance.

Each text was divided into four sections of approximately two sentences each. Participants read the first section silently and then hit the spacebar. A beep prompted a think-aloud response. To facilitate think-aloud behaviors, participants were provided with a list of 10 sentence starters (e.g., This is what is happening…, I wonder…, I predict…) that corresponded with the critical strategies. Think-aloud exemplars for a sample story were presented to familiarize the participants with the procedure. Once they had completed their first think-aloud, participants hit the spacebar to continue to the next section and earlier sections remained on the screen. When participants completed their final think-aloud, they pressed the spacebar to continue to the comprehension questions. Since the study's goal was to examine the association between text comprehension and strategy use (and not readers' ability to search the text for the correct answers), participants did not have access to the text for the comprehension questions. Participants were invited to complete the think-alouds in their preferred language.

Although each text from the GORT-4 had five multiple choice questions, researcher-generated open-ended comprehension questions were employed. Open-ended questions require readers to rely on their mental representation of the text to generate the answers (Collins et al., 2018). Additionally, they avoid the high cognitive load associated with comparing answers in a multiple-choice format (Collins et al., 2018). Each text had three questions that required either providing information found directly in the text (literal questions), generating a necessary inference (i.e., for text cohesion, a reader must make an inference that is not explicitly stated but is assumed by the author) or generating elaborative inference (an inference is made, but it is not necessary to understand the text). For example, in a text about the difficulties growing crops. Readers were asked the literal question “what did the farmers do to protect their crops?” the necessary inference question: “why were the farmers concerned about their crops? and elaborative inference question “how do you think the farmers feel?” To increase the likelihood participants understood and were able to respond to the questions, the examiner offered to read the questions aloud and participants were invited to respond in their preferred language.

#### Think-aloud data coding

Audio recordings of the think-aloud data were transcribed and coded for ten strategies (see Supplemental material for examples of each strategy in a think-aloud). Only one participant responded in a language other than English and their responses were translated to English. Each participant had four think-alouds per text and raters identified and tallied tokens of strategies in each think-aloud. Two raters met to calibrate their coding. To ensure coding remained consistent, think-alouds were examined in small batches (~10 participants at a time). In the child dataset, to verify the inter-rater reliability of the coding scheme, the second rater independently rated 30% (*N* = 48) of the participants. First and second raters' profiles of strategy use were compared (see below); However, the second rater reviewed the coding for all think-alouds, and the finalized coding was based on agreement from both coders. The procedure was slightly different for the adult data. Here, the primary rater's coding was verified by a second rater to finalize coding and a third rater independently coded a data subset (42%; 14 participants) for reliability.

To gain a profile of strategy use, frequency was calculated by tallying the number of times each strategy was identified for each participant. Inter-rater reliability was computed on the strategy profiles. Total count inter-observer agreement (Cooper et al., 2007) is the percentage agreement for each strategy (agreement/agreement + disagreement) averaged across each participant. Overall agreement was 80% in both the child and adult coding. Agreement of around 80% has been previously observed for think-aloud data (Chamot and El-Dinary, 1999). Finalized coding was based on the consensus of two raters. Importantly, the relative use of each strategy was captured by each rater. There was an average correlation of 0.89 between raters in the child data and 0.88 between raters in the adult data, indicating that raters were similarly distinguishing high users of a strategy from low users of a strategy.

Reading Comprehension responses were scored on a scale of 0 to 2 (*0 being incorrect, 1 being incomplete correct answer, 2 being complete correct answer)*. A scoring rubric was constructed for each question. For example, participants had to identify the two issues mentioned in the text about why farmers were concerned about crops (e.g., insects and weather) to receive two points. Identifying only one problem resulted in a single point. Similarly, in response to how farmers protect their crops, mentioning chemicals got a single point, stating that these chemicals were used to kill insects received two points. One rater initially scored each question, and their scoring was confirmed by a second rater. Disagreements were discussed and the rubric was refined if necessary and applied to all responses. Answers to the same question were compared directly to ensure similar responses were assigned the same scores. Responses from a randomly selected subset of participants (35%) were re-coded by a third rater using the rubric and 86% agreement was achieved. There were 3 questions per story with a potential maximum score of 24. A single score was generated since reading comprehension assessments regularly report a single score and include questions that require both literal and inferential information (Eason et al., 2012).

### Procedure

Ethics approval was obtained from the university's non-medical research ethics review board and subsequently by the school board research committee. Data was collected in the spring (at the end of the academic year) of two consecutive school years from two separate cohorts. Following consent, each participant completed testing sessions individually. For the children, testing occurred in their school in a quiet space and was conducted over two sessions for ~30 min each. In the first session, students did the vocabulary measure (i.e., PPVT) and the word reading fluency measure (i.e., TOWRE). In the second session, they completed the reading comprehension task. Of note, these tasks were part of a larger test battery in the schools. For the adults, the testing session took place at the University in a single session.

## Results

The descriptive statistics for language measures are reported in Table 1. All values are raw scores. A multivariate analysis of variance with receptive vocabulary, total word reading fluency and reading comprehension scores as dependent measures was conducted. The analysis met the assumptions for no multicollinearity. The Mahalanobis distance revealed one outlier that was subsequently removed from the multivariate analysis. Box' M and Levene's tests were significant, indicating that the assumption of normality was violated. To address this concern, Pillai's trace was used and a *p*-value of 0.001 was set for both the multivariate and univariate analyses (Allen and Bennett, 2008).

The multivariate analysis revealed an overall main effect of language group, *F*(6, 376) = 7.32, *p* < 0.001, $n_{p}^{2}$ = 0.11. Univariate ANOVAs (analysis of variance) revealed that group differences were present in the receptive vocabulary measure, *F*(2, 189) = 14.94, *p* < 0.001, $n_{p}^{2}$ = 0.14, and the reading comprehension measure, *F*(2, 189) = 9.10, *p* < 0.001, $n_{p}^{2}$ = 0.09, but not in total word reading fluency, *F*(2, 189) = 1.79, *p* = 0.171, $n_{p}^{2}$ = 0.02. Sheffe *post-hoc* comparisons found that for receptive vocabulary, monolingual children outscored both the bilingual children, *p* < 0.001, and the bilingual adults, *p* < 0.01. No differences were observed between bilingual groups on vocabulary knowledge, *p* = 0.75. In contrast, on the reading comprehension measure, no differences were observed between the monolingual children and the bilingual adults, *p* = 0.91. However, both groups outperformed the bilingual children, *p*s < 0.02. Of note, average reading comprehension scores of ~50% appear to be low, but do in fact reflect that, on average, participants were providing partially correct answers.

### Strategy recruitment

Table 2 reports the means, standard deviations, and medians for each strategy type by group. The distributions violated the assumption of normality and therefore Kruskal-Wallis H non-parametric tests were conducted on each strategy by language group. There were eight significant Kruskal-Wallis H tests: reference to vocabulary, χ^2^(2) = 7.11, *p* < 0.05, reference to text structure, χ^2^(2) = 23.57, *p* < 0.001, necessary inferencing, χ^2^(2) = 35.16, *p* < 0.001, elaborative inferencing, χ^2^(2) = 16.01, *p* < 0.001, connecting, χ^2^(2) = 23.99, *p* < 0.001, summarizing, χ^2^(2) = 6.84, *p* < 0.05, reference to background knowledge, χ^2^(2) = 6.98, *p* < 0.05, and predicting, χ^2^(2) = 6.84, *p* < 0.05. No main effect of group was observed in visualizing or questioning, *p*s > 0.05.

**Table 2:** Mean and median sums of strategy use as a function of language group.

**Strategies**	**Monolingual children**		**Bilingual children**		**Bilingual adults**	
	**Mean (SD)**	**Median**	**Mean (SD)**	**Median**	**Mean (SD)**	**Median**
Vocabulary	0.7 (1.7)	0.0	**1.8 (3.7)**	**0.0**	0.3 (0.5)	0.0
Text Structure	0.5 (1.0)	0.0	0.7 (1.5)	0.0	**1.9 (2.0)**	**1.0**
Necessary Inferencing	7.7 (5.4)	6.0	6.9 (5.5)	6.0	**14.3 (6.0)**	**14.0**
Elaborative Inferencing	7.0 (6.7)	5.0	7.4 (7.2)	5.0	**10.5 (4.6)**	**10**
Connecting	1.0 (1.3)	1.0	1.0 (1.5)	0.0	**2.7 (2.2)**	**2.0**
Summarizing	3.8 (4.1)	2.0	5.4 (5.7)	3.0	**5.7 (4.5)**	**5.0**
Background Knowledge	2.5 (3.6)	1.0	1.5 (2.5)	1.0	**2.3 (1.7)**	**2.0**
Predicting	**4.9 (4.7)**	**4.0**	3.1 (3.8)	2.0	4.0 (3.2)	4.0
Questioning	3.1 (4.8)	1.0	2.2 (3.6)	1.0	2.1 (2.6)	1.0
Visualizing	1.9 (3.6)	0.0	1.3 (2.7)	0.0	0.8 (1.6)	0.0

Bolded values indicate which strategies differed among groups and which group used the strategy significantly more than at least one other group.

*Post-hoc* comparisons using Bonferroni adjustments revealed that for reference to vocabulary, the bilingual children reported marginally greater use of this strategy than the bilingual adults, *p* = 0.07, but not significantly more than monolingual children *p* = 0.11. Adult bilinguals reported significantly more reference to text structure, *p*s < 0.001, as well as greater use of necessary inferencing, *p*s < 0.001, elaborative inferencing, *p*s < 0.01, and connecting, *p*s < 0.001, that both child groups; no differences were observed between child groups on these strategies, *p*s > 0.05. For summarizing, the bilingual adults reported this strategy significantly more often than the monolingual children, *p* < 0.05, but not the bilingual children, *p* > 0.05. For background knowledge, bilingual adults expressed more reference to background knowledge than bilingual children, *p* < 0.05; no other group differences were observed on background knowledge. For predicting, monolingual children reported marginally greater use than bilingual children (*p* = 0.06) but no difference from bilingual adults, *p* > 0.05.

### Predictors of reading comprehension success

Table 3 reports the bivariate correlations of reading comprehension with both language measures and strategies for each group and for the full sample. Partial correlations are also reported to examine whether the relationships between reading comprehension and strategy use remain when the influence of age, English receptive vocabulary and word reading are removed. For all groups, vocabulary correlated significantly with reading comprehension. Total word fluency correlated significantly with reading comprehension for the children only. Reference to text structure, connecting and necessary inferencing were all correlated to reading comprehension in each group. However, for the monolingual children, the effects disappeared for text structure and connecting when language measures and age were partialled out. Additionally, some differences across groups were observed. Elaborative inferencing was a significant correlate in the children but not the adults. Visualizing was significantly correlated for the monolingual children, whereas vocabulary and use of background knowledge were significant for the bilingual children in the partial correlations. Summarizing, questioning, and predicting were not significant partial correlates with reading comprehension for any groups. Taken together, as a full sample, most strategies correlated with reading comprehension to some degree, with the strongest correlations observed for inferencing behaviors, making connections within the text and commenting on text structure.

**Table 3:** Correlations of language measures and strategy use with RC scores for all three language groups.

**Measures**	**Monolingual children**		**Bilingual children**		**Bilingual adults**		**Full sample**	
	**Bivariate**	**Partial**	**Bivariate**	**Partial**	**Bivariate**	**Partial**	**Bivariate**	**Partial**
Age	0.39^**^	—	0.51^**^	—	−0.01	—	−0.02	—
**Language measures**								
Vocabulary knowledge	0.66^***^	—	0.77^***^	—	0.48^**^	—	0.73^***^	—
Word fluency total	0.65^***^	—	0.61^***^	—	0.12	—	0.55^***^	—
**Strategies**								
Text structure	0.26^*^	0.07	0.52^***^	0.39^***^	0.43^**^	0.42^*^	0.41^***^	0.33^**^
Connecting	0.33^**^	0.19	0.48^***^	0.34^**^	0.52^**^	0.47^**^	0.42^***^	0.32^***^
Necessary inferencing	0.47^***^	0.30^*^	0.66^***^	0.39^***^	0.50^**^	0.45^**^	0.55^***^	0.40^***^
Elaborative inferencing	0.52^***^	0.44^**^	0.55^***^	0.42^***^	0.18	0.33	0.49^***^	0.44^***^
Visualizing	0.26^*^	0.27^*^	0.20	−0.09	0.15	0.27	0.21^**^	0.11
Vocabulary	0.04	0.04	0.03	0.26^*^	0.28	0.23	−0.04	0.20^**^
Background knowledge	0.17	0.11	0.27^**^	0.29^**^	0.12	0.05	0.23^**^	0.18^*^
Summarizing	0.03	0.09	0.14	0.13	0.38^*^	0.33	0.09	0.17^**^
Questioning	0.31^*^	0.19	0.23^*^	0.18	−0.15	0.01	0.22^**^	0.17^*^
Predicting	0.14	0.23	0.16	−0.15	0.29	0.32	0.19^**^	0.04

* p < 0.05,

** p < 0.01,

*** p < 0.001. Bolded values denote significant correlations.

For the full sample, hierarchical linear regression analyses were performed to examine how strategy use accounted for reading comprehension beyond age, vocabulary knowledge and word reading fluency. To reduce the number of predictors, decrease any multiple collinearities and to determine the relationship between strategies themselves, a principal component factor analysis was conducted. The KMO measure of sampling adequacy value was 0.63 and deemed adequate. Four factors were generated that accounted for 65% of the variance. See Table 4 for the four-factor structure. Factor 1 consisted of making connections within the text and reference to text structure and necessary inferencing. It was called textbase strategies since these strategies involved making sense of the meaning units within the text itself. Factor 2 included reference to vocabulary, reference to background knowledge and questioning. This factor was named accessing knowledge since these behaviors involved accessing both lexical and semantic knowledge. It was often done in the context of questioning the text. Factor 3 was called elaboration as it pertained to elaboration both in terms of inferences but also in terms of creating visual imagery. Finally, Factor 4 (Prediction) consisted of prediction behaviors and failures to summarize. Here, readers were not engaged in behaviors grounded in the text but were anticipating upcoming events or information.

**Table 4:** Factors analysis components for the predictor variables.

**Construct**	**Text base**	**Accessing knowledge**	**Elaboration**	**Prediction**
Connecting	**0.86**	0.08	0.05	0.13
Text structure	**0.81**	0.09	−0.13	−0.03
Necessary inferencing	**0.64**	−0.24	0.44	−0.22
Vocabulary	0.08	**0.66**	−0.20	−0.20
Background knowledge	0.21	**0.65**	0.11	0.16
Questioning	−0.15	**0.62**	0.16	0.07
Elaborative inferencing	0.49	0.18	**0.60**	−0.04
Visualization	−0.12	0.03	**0.85**	0.02
Predicting	0.20	−0.14	0.05	**0.87**
Summarizing	0.37	−0.34	0.12	**−0.68**

Bolded values indicate that this variable loaded onto the corresponding factor.

Age, vocabulary, and word fluency were entered in the initial step of the hierarchical regression model (Core model) followed by the four strategy factors in the second step (Full model) using the enter input method. The model assumptions were met (e.g., appropriate sample size for number of predictors, no multicollinearity). Table 5 reports the core and full models. Both models were significant: core model, *R* = 0.76, *F*(3, 188) = 89.68, *p* < 0.001; full model, *R* = 0.85, *F*(7, 184) = 67.92, *p* < 0.001. The full model accounted for 71% of the variance in reading comprehension scores, an increase of 13.2% from the core model. The positive regression weights for vocabulary knowledge and word fluency remained when strategy use factors were input; age was no longer a significant predictor. Textbase, Accessing Knowledge and Elaboration were all significant positive predictors of reading comprehension scores with textbase strategies accounted for the most variance followed by Elaboration and then Accessing Knowledge.

**Table 5:** Reading comprehension regression models for the full sample.

**Predictors**	**b**	**SE**	**β**	***t***	**Sig**.
**Core model**				
Constant	−3.20	1.11		−2.88	0.004
Age	−0.015	0.005	−0.13	−2.66	0.008
Vocabulary knowledge	0.09	0.008	0.63	11.03	< 0.001
Word fluency	0.04	0.010	0.23	4.02	< 0.001
**Final model**				
Constant	−0.92	0.983		−0.94	0.35
Age	−0.001	0.005	−0.009	−0.21	0.83
Vocabulary knowledge	0.076	0.008	0.511	9.76	< 0.001
Word fluency	0.025	0.009	0.139	2.90	0.004
Textbase	1.65	0.219	0.340	7.52	< 0.001
Accessing knowledge	0.660	0.190	0.136	3.47	< 0.001
Elaboration	0.954	0.202	0.197	4.72	< 0.001
Prediction	−0.088	0.197	−0.018	−0.446	0.656

## Discussion

The present study investigated the relationships between reported strategy use, language knowledge and reading comprehension in three groups. Of interest was (1) how groups differed in their reported strategy use, (2) which strategies were associated with reading comprehension performance and (3) whether reported strategy use could explain unique variance in reading comprehension performance not explained by language variables. By including the three groups with different levels of language proficiency and reading experience, we gained insight into these variables' contributions to strategy use and reading comprehension success. We found that bilingual adults reported more inferencing behaviors, references to text structure and connecting behaviors than the children. Monolingual children made marginally more predictions than their bilingual peers. Finally, although receptive vocabulary knowledge and word reading fluency predicted reading comprehension scores, several strategy factors also accounted for unique variance in reading comprehension scores, with an emphasis on textbase strategies. Such findings highlight the contributions of strategic behaviors to reading comprehension success.

Our results are consistent with the SVR model (Hoover and Gough, 1990). Both word reading fluency and vocabulary were significant predictors of reading comprehension performance. Notably, word reading fluency was correlated with reading comprehension performance for the children only. This finding is consistent with work demonstrating that word reading abilities play a unique role earlier in reading comprehension development (e.g., Storch and Whitechurst, 2002; Proctor et al., 2006; Gunnerud et al., 2022) but less so for adults (Landi, 2010). However, increased word reading automaticity in the bilingual adults is an unlikely explanation since no differences in word reading fluency were observed among the three groups. A more likely possibility is that since our reading task was not speeded, adults modulated their reading speed better than the children.

Higher English receptive vocabulary was associated with better reading comprehension outcomes for all groups. This finding was consistent with previous research (e.g., Kendeou et al., 2009; Babayigit and Shapiro, 2020; Raudszus et al., 2021). Receptive vocabulary was among the strongest correlates of reading comprehension success for each group and as expected was the best predictor in the regression model. Of note, consistent with Melby-Lervåg and Lervåg's (2014) meta-analysis, vocabulary knowledge was a stronger predictor in for the bilingual children in their L2 than the monolingual children in their L1. Given that overall, our bilingual children had less English vocabulary knowledge, it is not surprising that the monolingual children produced higher reading comprehension scores; less L2 vocabulary knowledge makes reading comprehension tasks more challenging for second language learners (Melby-Lervåg and Lervåg, 2014).

In general, the monolingual and bilingual children reported similar strategies with some subtle differences. Monolingual children tended to state more predictions, whereas the bilingual children made more references to vocabulary words. Notably, these results are consistent with Frid and Friesen (2020) who observed that French Immersion students reported more predicting in their dominant language and referred more to vocabulary words in their non-dominant language. Jiménez et al. (1996) noted that identifying vocabulary words and summarizing are often recruited more in a less proficient language. Nonetheless, the overall similar pattern of reported strategy use among groups suggests that the main reason for the reading comprehension differences was due to English language proficiency, where receptive English vocabulary was used as a proxy here.

A comparison of the bilingual children and adults on language measures provides insights into differences in reading comprehension success. Despite being older, the bilingual adults did not differ from bilingual children on receptive English vocabulary knowledge but did outperform the bilingual children on reading comprehension. The lack of difference in English receptive vocabulary is likely because the bilingual children had spent significantly more time in an Anglophone community than the bilingual adults. The adults, in contrast, had significantly more overall literacy experience. They learned to read in their L1 and could use reading strategies to offset their lack of L2 knowledge. The benefit of strong L1 abilities has been originally detailed by Cummins (1981) in his linguistic interdependence hypothesis. Higher-order reading strategies learnt in the L1 can be used in L2 (assuming a minimum level of L2 proficiency). This common underlying proficiency presumes that academic competencies are shared across languages and that individuals can draw on higher-order skills such as analysis, integration, and reasoning in both languages. In contrast, the bilingual children were primarily developing reading skills and higher-order strategies in their L2 (as opposed to their L1) alongside their monolingual peers.

An examination of reported strategy use provides insights into why bilingual adults outscored the bilingual children on reading comprehension. The bilingual adults reported significantly more necessary inferences, elaborative inferences, reference to text structure and connecting behaviors than both groups of children. Importantly, these behaviors were correlated with reading comprehension in each group, providing evidence that overall greater use of these strategies likely facilitated reading comprehension in the bilingual adults. Likewise, these strategies were critical in accounting for unique variance in reading comprehension performance in the full sample. Of note, in Friesen and Frid (2021) when presented with challenging texts at and above their reading levels, English-French bilingual adults also greatly relied on making inferences. Unlike the current study, they also tended to favor more summarizing statements; likely to confirm their understanding.

Taken together, these behaviors are necessary to construct a comprehensive mental representation of the text. As described in the Construction-Integration Model (Kintsch, 2005), readers need to generate and select relevant inferences, and then integrate these meaning units by making connections. Additionally, the ability to identify the text structure enables a reader to create a scaffold on which to insert newly generated information (Gernsbacher et al., 1990; Cain, 2010). Readers who are aware of the text structure can anticipate upcoming information and then organize the information for later retrieval. A clear mental representation of the text involves understanding the relationship between ideas and organizing these ideas; doing so, enables better retrieval, and consequently, better reading comprehension performance.

Despite some overall group differences, there was also variability in reading comprehension performance within each language group. From an educator's perspective, an understanding of which strategies are associated with reading success may be sufficient for the classroom. The bivariate correlations in Table 3 provide insight into the likelihood that students will be successful on a subsequent reading test. Indeed, it was making connections, generating inferences and reference to text structure that are all markers of subsequent reading comprehension success for all readers. Looking for these behaviors during independent or guided reading may serve as a diagnostic or formative assessment of effective strategy use as readers build toward reading comprehension success. Importantly, individual strategies are not used in isolation and may be associated with each other in readers' repertoires (Frid and Friesen, 2021). Thus, isolating significant strategy use demonstrates that strategy use accounts for reading success beyond what is accounted for by language measures. This is important given previous work has failed to isolate unique contributions of strategy use and language ability (e.g., Lin and Yu, 2015; García and Godina, 2017). Here we observed that strategy use accounted for significant variance on top of the language measures.

Inferencing behavior was a significant correlate of reading comprehension performance. The robust nature of these findings highlights the importance of generating inferences as part of developing a situation model (i.e., a meaning-based representation that links text content to the reader's previous knowledge). Indeed, previous work has isolated offline inferential abilities as predictors of reading success (e.g., Oakhill et al., 2003; Ahmed et al., 2016). Raudszus et al. (2019) reported that the ability to build a situation model accounted for significant variance in reading comprehension beyond linguistic and cognitive predictors in both bilingual and monolingual Grade 3 students. Here we demonstrate that greater articulation of inferences during reading is also associated with better performance on a subsequent test that necessitates this inferential knowledge. Importantly, directly teaching inferential skills has been shown to improve reading comprehension performance (Silverman et al., 2014). Silverman et al. (2014) found that teachers' use of instruction that targeted inferential comprehension was positively associated with reading comprehension gains in both monolinguals and bilinguals. Importantly, students should be taught how to engage in effective think-alouds to promote effective strategy use (Kim and Cha, 2015; Friesen and Haigh, 2018) and consolidate their inferences into long-term memory.

In the regression analysis, three of the four strategy factors were positively related to reading comprehension performance. Like previous research (Frid and Friesen, 2020), the factor associated with building a mental representation of the text through constructing and integrating meaning units (i.e., textbase) was the second strongest predictor of reading comprehension performance after vocabulary knowledge. Elaborative behaviors and accessing knowledge were also associated with reading comprehension success but to a less extent. Finally, predicting behaviors were not particularly beneficial. These finding confirms previous work (i.e., Duke and Pearson, 2009; Frid and Friesen, 2020), wherein predicting by itself was not a significant predictor of reading comprehension success in children. Friesen and Frid (2021) reported that with adults, there is a greater tendency to make both predictions and then explicitly connect back to their predictions; this behavior is associated with greater reading comprehension success.

### Implications

Our results demonstrate that reported strategy use is associated with reading success beyond language knowledge. Of interest to educators is how to support the development of these skills alongside language instruction. Given the rising numbers of English language learners in schools (Census Canada, 2017), an important avenue of future research will be to understand how strategy use and language proficiency develop interactively throughout schooling. Since English vocabulary knowledge was strongly related to reading comprehension success for all readers, continued language and vocabulary development should facilitate reading comprehension success (see Babayigit and Shapiro, 2020). However, explicit instruction on how to utilize strategies together to build a mental representation of the text is clearly warranted, particularly given the variability in strategy use and reading comprehension performance within all groups. One suggestion would be to jointly work on gaining language proficiency and strategy use by scaffolding language instruction (e.g., guided reading) to focus directly on strategy development.

A few considerations become key when determining what strategies to teach to support reading comprehension performance. Our research and previous research have found that questioning (e.g., Yopp, 1988), visualization (e.g., Pressley, 2000), and reliance on text structure (Oakhill et al., 2003) are all associated with greater comprehension success. However, our work demonstrated that these strategies were less frequently reported and as such may have served as markers of comprehension rather than fully realized strategies in the readers' repertoires (see also Frid and Friesen, 2020 and Friesen and Frid, 2021). Consequently, there may have been unrealized strategies that would have increased comprehension performance that readers in general fail to report or to use. For educators, assessing which strategies each individual student is using becomes essential to understanding which strategies require additional support and which strategies require direct instruction (Friesen and Haigh, 2018).

Our implications should be considered in light of the study's limitations. The correlational nature of the current study makes caution necessary in recommending specific strategies to teach based on correlations or regression models. Likely, there is a bidirectional relationship between reading comprehension performance and reported strategy use, such that a reader's comprehension dictates the strategies that they can report. But in return, the selection of effective strategies consolidates content in memory. Importantly, the act of doing a think-aloud may increase processing beyond what is expected during silent reading. For some readers, this opportunity may be beneficial for reading comprehension. However, for others doing think-alouds may negatively impact comprehension, particularly if respondents were not employing strategies that supported consolidating content in memory. Future research could examine how strategy use reported in think-alouds are related to performance on a different reading comprehension assessment as this approach would reduce concerns about the think-alouds impacting the assessment of reading comprehension ability.

Another concern is that a reader may choose to only report a subset of their thoughts due to perceived time constraints. For example, one possibility is that more successful comprehenders are providing elaborative inferences because they have the foundational skills (e.g., summarizing, drawing necessary inferences) required to think beyond the text and consequently elaborative inferencing stands as the representative of a group of strategies. Thus, it is likely a constellation of strategic behaviors that support comprehension. Indeed, findings from both the current study as well as Frid and Friesen (2021) imply that utilizing strategies in concert is particularly effective in generating a comprehensive mental text representation upon which to base reading comprehension performance.

An additional consideration for teachers is the selection of reading comprehension test format and what knowledge or skills in addition to reading comprehension that it may be assessing. Unfortunately, research has demonstrated that even with standardized measures, only moderate correlations exist between test formats (Keenan and Meenan, 2014) and thus tests tap into different underlying skills in addition to reading comprehension (Spencer et al., 2019). For example, Carlisle and Rice (2004) found that cloze test performance is associated with semantic understanding at the sentence level rather than at the text-level. Given that preferred reading comprehension questions require students to draw on a deeper understanding of the text (Spencer et al., 2019), we selected to use open-ended questions that required drawing conclusions to be successful, and subsequently found that reliance on inferencing, connecting, reference to text structure was particularly fruitful for our reading comprehension measure. It remains to be seen if reliance on these strategies would be as successful with other response formats. Ideally, educators should be mindful of the alignment between the reading comprehension strategies they are teaching and how they are assessing students' understanding.

In conclusion, the current study was able to identify reported strategies that underlie successful reading comprehension in two ways. This was accomplished, first, by examining strategy differences between groups that were matched on either vocabulary knowledge or reading comprehension performance. Secondly, this question was addressed by examining which strategies were associated with reading comprehension performance and which strategies accounted for unique variance beyond vocabulary knowledge, age and word reading fluency. Strategies that enabled readers to identify implicit meaning (i.e., inferences), integrate meaning across the text (i.e., connections) and organize their knowledge (i.e., reference to text structure) served readers best in encoding and retrieving knowledge.

## Data availability statement

The datasets presented in this article are not readily available because at the request of the participating school board, guardians' consent was not obtained to share their child's datasets widely. Requests to access the datasets should be directed to DF; Deanna.Friesen@uwo.ca.

## Ethics statement

The studies involving human participants were reviewed and approved by Western University: Non-Medical Ethics Review Board. For the child participants, written informed consent to participate in this study was provided by the participants' legal guardian/next of kin. Adult participants provided written informed consent.

## Author contributions

DF, KS, and TA contributed to conception and design of the study. A subset of children's data was collected and analyzed by KS for their MA Thesis. The adult data was collected and analyzed by TA for their MA thesis. DF and AC developed the coding scheme. AC coded the majority of the data. DF conducted the combined statistical analyses and wrote the first draft of the manuscript. All authors contributed to manuscript revision, read, and approved the submitted version.

## Funding

This research was supported by a Social Sciences and Humanities Research Board Seed Research Grant from Western University. It was also supported by an Social Sciences and Humanities Research Council of Canada Insight grant (435-2018-442).

## Conflict of interest

The authors declare that the research was conducted in the absence of any commercial or financial relationships that could be construed as a potential conflict of interest.

## Publisher's note

All claims expressed in this article are solely those of the authors and do not necessarily represent those of their affiliated organizations, or those of the publisher, the editors and the reviewers. Any product that may be evaluated in this article, or claim that may be made by its manufacturer, is not guaranteed or endorsed by the publisher.
