# Supplementary material for: Reading comprehension and strategy use: Comparing bilingual children to their monolingual peers and to bilingual adults
Source: Front Psychol. 2022 Nov 24;13:986937. doi: 10.3389/fpsyg.2022.986937 (PMC9730700; doi:10.3389/fpsyg.2022.986937)
Supplement: Supplementary file 1 [file Table_1.DOCX]

Supplementary Materials: Reading Strategies Descriptions and Examples

| Strategies | Definition | Examples (taken from Participants’ think alouds) |
| --- | --- | --- |
| Vocabulary | Referring to a vocabulary word because it was difficult to understand.  The reader may also refer to the vocabulary word to point out the significance of the word. | “There are some unfamiliar words so I may have some troubles understanding that (the text). **There are some words like robot.”** |
| Text Structure | Referring to the layout of the text (e.g. noticing that the text is a story). Mentioning the intent of the author or commenting on how an idea is expressed that implies the structure of the text. | “**It seems like a story about** how Winnie saved herself and her brother from a storm.”  “I think **it is a happy ending** because when she was exhausted there was some food that emerged from the darkness.” |
| Summarizing | Paraphrasing the text or identifying the main ideas of the text by re-stating them. | “This means the **farmers all over the world are facing many problems** like the crops are attacked by insects and some disease.” (*repeating information from the story*) |
| Necessary Inferencing | Identifying additional information that is needed to understand the text. This information is not found in text so it is “reading between the lines”. | “So, if there is a little rain this year **then the plants will not grow very well.”** (*not present in the story*)”  “I think this means that **the north place is a good place or is a free place for the slaves**.” *(in response to information about slaves heading north)* |
| Elaborative Inferencing | Going beyond the text and identifying new information that is not necessary to understand the text. (e.g. commenting on the personality traits of a character) | “I think the turtle may be **really lonely.**” *(in response to having no friends)*  “I think that what Harriet did was something that not many people could do, what **she did was really brave** and very dangerous for her and she decided to do it.” |
| Predicting | Making guesses on what might occur next in the text. | **“I predict that this turtle wants to make friends with this eagle** and want to chat with her.”  “Through this atmosphere, **I predict that all the farmers will very disappointed and maybe they will give up to being a farmer or want to change their job.”** |
| Connecting | Referring to an earlier part of the text. Referring to a previous think-aloud which may have included a prediction. | **“My guess is right.** The turtle fell straight into the ocean because she opened her mouth.” |
| Visualizing | Forming a mental image of the text or information not in the text that helps the reader understand the text. | “I imagine that there is a little turtle. **I am picturing the turtle on the eagle’s back.”**  **“I am picturing the wanted wall**. (for Harriet)” |
| Questioning | Questioning the information in the text or questioning the meaning of a statement. Such as questions about who, what, where, when and how. | **“I wonder** how she could run away to the north? How many difficulties she has experienced? Wont she be afraid of being caught by her owner?”  **“I wonder** who Harriet is, is she someone like political or someone who want to set up a revolution?” |
| Background Knowledge | Referring to information that the participant has previously learned or information that is related to their life and life experiences in order to better understand the text. | “**This makes me think of my father**, my father also has a farm. He plants many potatoes and other plants.”  “I think **this maybe come from a book talking about slaves** or a movie talking about how to free slaves.” |
